# Myocardial ischaemia reperfusion injury and cardioprotection in the presence of sensory neuropathy: Therapeutic options

**DOI:** 10.1111/bph.15021

**Published:** 2020-03-21

**Authors:** Péter Bencsik, Kamilla Gömöri, Tamara Szabados, Péter Sántha, Zsuzsanna Helyes, Gábor Jancsó, Péter Ferdinandy, Anikó Görbe

**Affiliations:** ^1^ Cardiovascular Research Group, Department of Pharmacology and Pharmacotherapy, Faculty of Medicine University of Szeged Szeged Hungary; ^2^ Pharmahungary Group Szeged Hungary; ^3^ Department of Physiology, Faculty of Medicine University of Szeged Szeged Hungary; ^4^ Department of Pharmacology and Pharmacotherapy, Medical School University of Pécs Pécs Hungary; ^5^ Molecular Pharmacology Research Group, Centre for Neuroscience, János Szentágothai Research Centre University of Pécs Pécs Hungary; ^6^ Department of Pharmacology and Pharmacotherapy Semmelweis University Budapest Hungary

## Abstract

During the last decades, mortality from acute myocardial infarction has been dramatically reduced. However, the incidence of post‐infarction heart failure is still increasing. Cardioprotection by ischaemic conditioning had been discovered more than three decades ago. Its clinical translation, however, is still an unmet need. This is mainly due to the disrupted cardioprotective signalling pathways in the presence of different cardiovascular risk factors, co‐morbidities and the medication being taken. Sensory neuropathy is one of the co‐morbidities that has been shown to interfere with cardioprotection. In the present review, we summarize the diverse aetiology of sensory neuropathies and the mechanisms by which these neuropathies may interfere with ischaemic heart disease and cardioprotective signalling. Finally, we suggest future therapeutic options targeting both ischaemic heart and sensory neuropathy simultaneously.

**LINKED ARTICLES:**

This article is part of a themed issue on Risk factors, comorbidities, and comedications in cardioprotection. To view the other articles in this section visit http://onlinelibrary.wiley.com/doi/10.1111/bph.v177.23/issuetoc

**Abbreviations**
AMI: acute myocardial infarction
CAN: cardiovascular autonomic neuropathy
CMT: Charcot–Marie–Tooth disease
FD: familial dysautonomia
HFpEF: heart failure with preserved ejection fraction
HSANs: hereditary sensory and autonomic neuropathies
IKBKAP: in B‐cells kinase complex‐associated protein
PDE5: PDE type 5
RA: rheumatoid arthritis
RTX: resiniferatoxin
SERCA2a: sarco‐endoplasmic reticulum Ca^2+^‐ATPase type 2a
SLE: systemic lupus erythematosus
SP: substance P
SST: somatostatin
STZ: streptozotocin
TH: tyrosine‐hydroxylase
TRP: transient receptor potential
TRPA1: transient receptor potential ankyrin 1
TRPV1: transient receptor potential vanilloid type 1

## 1 INTRODUCTION—CARDIOPROTECTION AND SENSORY NERVES IN THE HEART

The acute care of myocardial infarction has been dramatically improved by the introduction of percutaneous coronary intervention and stenting thereby decreasing its early mortality. However, the incidence of chronic postinfarction heart failure is rising faster than previously expected (Lam, Donal, Kraigher‐Krainer, & Vasan, 2011). Cardioprotective interventions such as local and remote ischaemic and pharmacological pre‐conditioning, post‐conditioning, and per‐conditioning have been discovered and extensively studied in the last three decades (Ferdinandy, Hausenloy, Heusch, Baxter, & Schulz, 2014). However, their clinical translation has been failed so far, most probably due to several confounding factors including co‐morbidities, co‐medications and variable genetic background (see for a recent extensive review Hausenloy et al., 2017). Among cardiovascular co‐morbidity states such as hyperlipidaemia, diabetes mellitus and confounders such as aging or sex differences (see for reviews Ferdinandy et al., 2014; Ferdinandy, Szilvassy, & Baxter, 1998), sensory neuropathy has also been recognized as a confounding factor. Nevertheless, only a small amount of preclinical and clinical data are available on the influence of sensory neuropathy to ischaemic heart disease and cardioprotection (Ieda & Fukuda, 2009).

**FIGURE 1:**
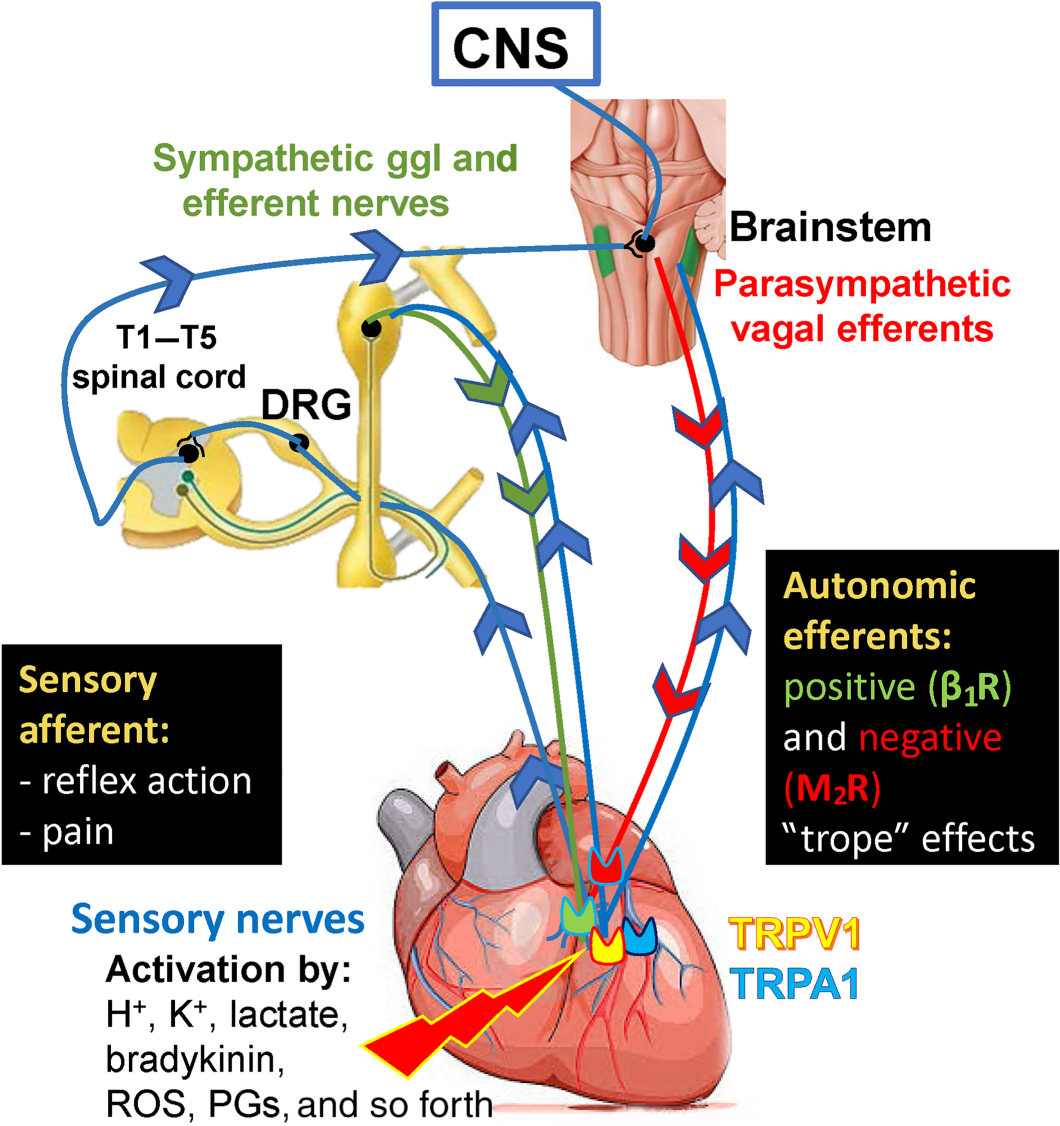
Sensory innervation of the heart. We focused on cardiac sensory nerves, but there are several other types of cardiac nerves and neurons (see in detail in the text of Section 2). Cardiac sensory fibres are coupled to both sympathetic and parasympathetic (vagus) nerves and can be separated from the autonomic nervous system. The capsaicin‐sensitive sensory nerve terminals are activated and sensitized by a variety of mediators produced by ischaemia, inflammation and tissue damage, and mediate sensory input pain and reflexes (classical afferent function). β1R, β_1_‐adrenoceptor; DRG, dorsal root ganglion; PGs, prostaglandins; M_2_R, M_2_ receptor; TRPA1, transient receptor potential ankyrin 1; TRPV1, transient receptor potential vanilloid‐1

The innervation of the heart is composed of intricate feedback loops of autonomic nervous system consisting of intrinsic cardiac ganglia, extracardiac intrathoracic ganglia, the spinal cord and areas of the CNS (Ardell et al., 2016). The effector machinery involves autonomic neurons of the sympathetic and parasympathetic nervous systems, as well as non‐adrenergic–non‐cholinergic (NANC) sensory nerves activated by hypoxia, ROS and increased concentrations of H^+^ and K^+^, leading to the release of neuropeptide transmitters (Franco‐Cereceda & Lundberg, 1988; see Figure 1). Cardiac sensory nerves can be localized separately having their own separate afferent soma inside intrinsic cardiac ganglionated plexi, but most of them are coupled to autonomic nerves including the parasympathetic vagal and sympathetic efferents (Ardell et al., 2016). In the clinical settings, pathologies impairing sensory nerves frequently also affect autonomic nerves, thereby combining the symptoms derived from both sensory and/or autonomic neuropathies, which lead to complex clinical presentation of the symptoms. Even in cases of primary, hereditary neuropathies (see below), neural injury shows a mixture of sensory and autonomic dysfunctions. Only preclinical models (e.g. capsaicin or resiniferatoxin (RTX)‐induced desensitization) are appropriate to clearly describe the cardiac effects of selective sensory neuropathy. Sensory neuropathy may affect all types of sensory neurons involved in myocardial regulation and therefore, in most pathologies affecting the complex cardiac sensory machinery, it is hard to distinguish between the localization of the lesion/s and the degree of neural damage.

Although sensory and autonomic neuropathies are important common contributors to the development of ischaemic heart disease, they are still not the focus of cardiovascular research. However, better understanding of these mechanisms may help to develop effective cardioprotective therapies. In this review, we collected the available data to summarize the diverse causes of sensory neuropathy and its contribution to cardiac pathology. We systematically evaluate preclinical models used to investigate the cardiac consequences of sensory neuropathy, as well as clinical data on concomitant occurrence of sensory neuropathies and cardiac pathologies. Furthermore, we summarize the mechanisms and effects of sensory neuropathy on myocardial ischaemia and cardioprotection. Finally, we highlight the therapeutic perspectives to achieve cardioprotection in the presence of sensory neuropathy.

## 2 PHYSIOLOGICAL ROLE OF CARDIAC SENSORY NERVES

The complexity of the functional role of sensory nerves innervating the heart has only recently been recognized. Classical studies have revealed the anatomy and functional significance of cardiac sensory nerves in the mechanisms of fundamental reflexes regulating heart function (Dawes & Comroe, 1954). Cardiac innervation includes a complex multiple‐levelled regulatory system consisting of feedback and feed‐forward neuronal circuits. Intrinsic cardiac ganglion plexus contains sympathetic and parasympathetic postganglionic efferent neurons, local circuit and afferent neurons, while extracardiac intrathoracic ganglia possess afferent and local circuit neuron as well as sympathetic postganglionic efferents. Neurons in both circuits form regulatory loops with higher centres (e.g. spinal cord, medulla oblongata and hypothalamus) under cortical neuronal control (Ardell et al., 2016). Cardiac afferents are coupled to both sympathetic and parasympathetic (vagus) efferent nerves and can also be separated from the autonomic nervous system (Figure 1). Cardiac afferent nerves, which can be excited by capsaicin, were first identified in investigations into the mechanisms of action of cardiorespiratory chemoreflexes. These studies showed that a substantial population of chemosensitive afferent nerves that convey these reflexes are activated by capsaicin (Porszasz, Gyorgy, & Porszasz‐Gibiszer, 1955). The characteristic cardiorespiratory chemoreflex evoked by capsaicin consisting of bradycardia, hypotension and apnoea could be inhibited by prior perineural treatment of the cervical vagal nerves with capsaicin, resulting in the selective interference of the function of capsaicin‐ but not phenylbiguanide‐sensitive C‐fibres (Jancso & Such, 1983). Chemosensitive afferents sense cardiac pain and mediate the cardiogenic sympathetic reflex (Pan & Chen, 2004).

Capsaicin is the pungent component of peppers that is able to selectively activate and/or disrupt an important subpopulation of sensory neurons, thinly myelinated Aδ and/or unmyelinated C fibres (Jancsó, 1968). Systemic administration of high‐dose capsaicin disrupt these primary sensory neurons in a rather more complex way. In newborn animals, systemic administration of high‐doses of capsaicin results in a selective loss of small, type B nociceptive primary sensory neurons (Jancso, Kiraly, & Jancso‐Gabor, 1977), but in adults, it produces long‐term desensitization and functional impairment of these neurons. Quantitative electron microscopic examination revealed a significant and selective loss of about 40% of unmyelinated afferent axons in peripheral sensory nerves (Jancso, Kiraly, Joo, Such, & Nagy, 1985) and up to 94% in spinal dorsal roots (Nagy, Iversen, Goedert, Chapman, & Hunt, 1983). Importantly, autonomic nerve fibres are not affected by capsaicin treatment (Jancsó, Such, Király, Nagy, & Bujdosó, 1985). In contrast to the comparatively moderate loss of C sensory fibre number, profound reduction was detected in the levels of neuropeptide transmitters specific to these nociceptive primary sensory neurons (Gamse, Lackner, Gamse, & Leeman, 1981; Jancso & Knyihar, 1975).

**FIGURE 2:**
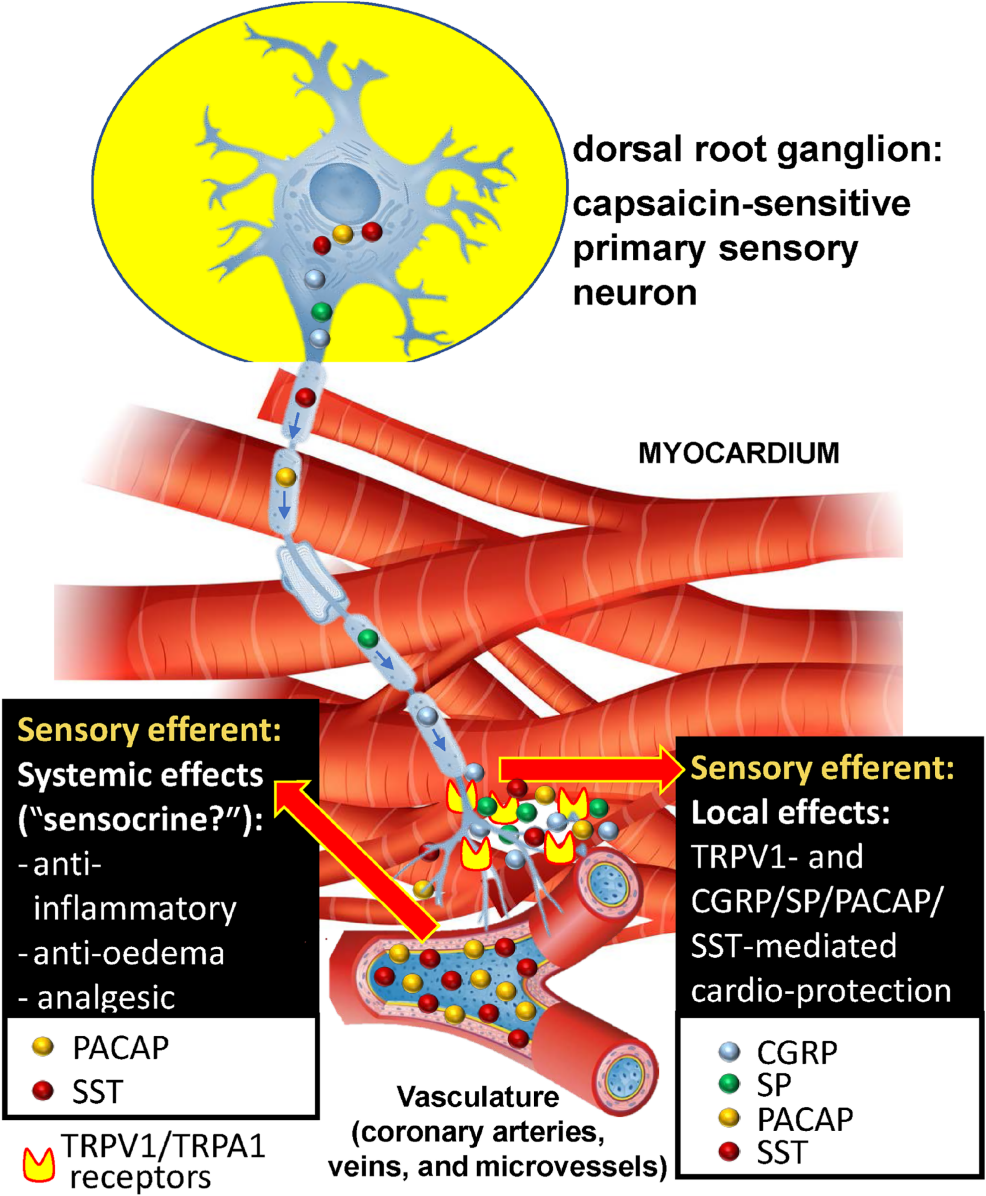
Sensory neuropeptides released from these activated fibres exert important functions on the heart both locally (local efferent function) and via the bloodstream (systemic efferent function). CGRP, substance P and PACAP induce vasodilatation, plasma protein activation, and immune cell activation in the innervated area collectively called neurogenic inflammation, while inhibitory mediators, such as SST and PACAP, also released from the same fibres exert anti‐oedema, anti‐inflammatory and analgesic actions after getting into the systemic circulation even at distant parts of the body. PACAP and CGRP are multi‐functional peptides; they are potent vasodilators but inhibit inflammatory cells and have cytoprotective actions. The cardioprotective role of the capsaicin‐sensitive sensory nerves is likely to be related to the protective neuropeptides, but the mechanism of action needs further investigations. PACAP, pituitary adenylate cyclase‐activating polypeptide; SP, substance P; SST, somatostatin; TRPA1, transient receptor potential ankyrin 1; TRPV1, transient receptor potential vanilloid‐1

There is ample evidence that the effects of capsaicin are being mediated through the activation of the transient receptor potential vanilloid type 1 receptor (TRPV1) also known as the vanilloid receptor 1 or the capsaicin receptor (Caterina et al., 1997) expressed in chemosensitive primary sensory neurons. TRPV1 receptor physiologically can be activated and/or sensitized by several stimuli, such as H^+^, K^+^, bradykinin, ROS and prostaglandins (Nagy, Santha, Jancso, & Urban, 2004; Randhawa & Jaggi, 2015; Figures 1 and 2). Another TRP receptor, transient receptor potential ankyrin 1 (TRPA1) is expressed and co‐localized with TRPV1 receptors in the cardiac muscle (Andrei, Sinharoy, Bratz, & Damron, 2016). TRPA1 is also present on vascular smooth muscle cells and sensory nerves in the heart, which are also activated by ROS, Ca^2+^ and prostaglandins (Wang, Ye, et al., 2019; Figures 1 and 2). Anatomical and functional evidence indicate that capsaicin‐sensitive primary afferent neurons of both spinal (thoracic dorsal root ganglia) and vagal (nodose ganglion) origins innervate the heart (Ferdinandy et al., 1997; Jancso & Such, 1983; Pan & Chen, 2004).

A significant proportion of chemosensitive afferents expressing the TRPV1 receptor are peptidergic, which are involved in more functions than just sensory input such as pain and reflex regulation of the cardiorespiratory system. Sensory neuropeptides are released from these fibres in response to activation not only experimentally by capsaicin and exogenous vanilloid, but also by a broad range of inflammatory stimuli and tissue irritants which exert important effector functions on the heart (Jancsó, 1968; Jancso, Kiraly, Such, Joo, & Nagy, 1987). CGRP, substance P (SP) and pituitary adenylate cyclase‐activating polypeptide (PACAP‐38) induce vasodilatation, plasma protein activation and mainly immune and inflammatory cell activation in the innervated area, collectively called neurogenic inflammation (Figure 2; Holzer, 1988). Meanwhile besides the pro‐inflammatory peptides, inhibitory mediators, such as somatostatin, galanin and opioid peptides are also released from the same capsaicin‐sensitive sensory nerves in response to activation, which have anti‐oedema, anti‐inflammatory, analgesic and cytoprotective actions when entering the systemic circulation, even at distant parts of the body (Holzer, 1988; Jancso et al., 1987; Szolcsanyi, Pinter, Helyes, & Petho, 2011). Pituitary adenylate cyclase‐activating polypeptide (Harmar et al., 2012) and CGRP (Li & Peng, 2002) are interesting as they are multifunctional peptides. They have strong vasodilating actions contributing to the vascular components of the inflammatory response, but also have a potent inhibitor action on the cellular inflammatory mechanisms and direct cytoprotective actions on the cardiomyocytes (Figure 2).

## 3 MOLECULAR CHANGES IN THE MYOCARDIUM IN THE PRESENCE OF CAPSAICIN‐INDUCED SENSORY NEUROPATHY

A limited number of preclinical papers are available in the literature which discuss the effect of sensory neuropathy on cardiac cell signalling. Most of the data are restricted to the sensory neuropathy evoked by systemic capsaicin treatment‐induced sensory desensitization. Therefore, in the following section, we will present data from papers using preclinical model of capsaicin‐induced sensory neuropathy.

More than a decade ago, Zvara et al. showed cardiac functional alterations and gene expression changes in rat hearts 7 days after the induction of sensory neuropathy by systemic capsaicin treatment, using DNA microarray technique. Capsaicin‐induced sensory neuropathy resulted in cardiac dysfunction characterized by elevation of left ventricular end‐diastolic pressure which led to significant up‐regulation of 47 and significant down‐regulation of 33 genes out of 6400 genes available on the microarray. Sensory neuropathy has been shown to influence a variety of neuronal and non‐neuronal genes in the heart including vanilloid receptor‐1 TRP protein, GABA receptor rho‐3 subunit, cytochrome P450 subfamily 2A, polypeptide 1 (CYP2A1), 5‐HT_3B_ receptor, NK_2_ receptor, matrix metalloproteinase‐13, farnesyl‐transferase, Apo B apolipoprotein, leptin and endothelial NOS (Zvara et al., 2006).

Bencsik et al. found that sensory neuropathy (chemo‐denervation) induced by 7 days of capsaicin treatment decreased the cardiac NO availability via decreased activity of Ca^2+^‐dependent NOS isoforms (endothelial NOS and nNOS) and increased SOD activity thereby lead to decreased basal ONOO^−^ formation and a reduction of S‐nitrosylation of sarco‐endoplasmic reticulum Ca‐ATPase type 2a (SERCA2a), which caused impaired myocardial relaxation in the rat heart. The gene expression pattern of the three different isoforms of NOS in the heart were also examined by real‐time quantitative PCR. Endothelial NOS (eNOS) was significantly down‐regulated due to capsaicin‐desensitization. However, the expression of neuronal and inducible NOS isoforms was unaffected (Bencsik et al., 2008).

A recent animal study of capsaicin‐induced sensory neuropathy revealed microRNA changes in the rat heart after 7 days of treatment (Bencsik et al., 2019). Echocardiographic data showed a moderate diastolic dysfunction in sensory neuropathy group and several microRNA showed altered expression. Out of 711 miRNAs represented on the miRNA microarray, the expression of 257 miRNAs was detectable, from which miR‐344b, miR‐466b miR‐98, let‐7a, miR‐1, miR‐206 and miR‐34b showed down‐regulation and miR‐181a was up‐regulated due to sensory neuropathy. The altered miRNAs were selected for an unbiased bioinformatic miRNA‐target network analysis, which predicted several target genes including insulin‐like growth factor 1, solute carrier family 2, facilitated glucose transporter member 12, eukaryotic translation initiation factor 4e and unc‐51 like autophagy activating kinase 2, which were validated as potentially contributing factors to cardiac diastolic dysfunction induced by sensory neuropathy (Bencsik et al., 2019).

## 4 THE EFFECTS OF SENSORY NEUROPATHY ON MYOCARDIAL ISCHAEMIA/REPERFUSION INJURY AND INFARCTION

The number of available published papers in this special cardiovascular field is quite limited. The first report for the effects of sensory neuropathy during the course of myocardial infarction was published by Nesto and colleagues. They showed that diabetes‐induced sensory neuropathy may contribute to asymptomatic myocardial infarction, probably due to lack of pain sensation as a result of injured sensory nerves (Nesto & Phillips, 1986). Subsequently, several publications confirmed the potential influence of diabetes‐induced sensory neuropathy in asymptomatic or silent ischaemia by enrolling high number of acute myocardial infarction (AMI) or coronary artery disease patients (Elliott et al., 2019). The first preclinical characterization of experimental acute myocardial ischaemia/reperfusion injury ex vivo after in vivo capsaicin‐induced sensory neuropathy was published in 1997 by Ferdinandy et al. The authors did not observe significant changes in ischaemia/reperfusion injury but a loss of pre‐conditioning‐induced cardioprotection (see next chapter). In another in vivo study of myocardial infarction in the presence of capsaicin‐sensitive sensory nerve degeneration (Zhang, Guo, Wang, & Wu, 2012), the authors used a rat model of capsaicin‐sensitive sensory desensitization induced by single subcutaneous capsaicin injection within the first 48 hr after birth. Selective desensitization of capsaicin‐sensitive nerves was validated by a significant deterioration in tail flick test in the capsaicin‐treated group of rats at12 weeks of age before conducting permanent coronary ligation. They found that infarct size was significantly increased by sensory desensitization 6 hr after the induction of coronary ligation. Furthermore, their finding was further supported by the increased troponin I level and enhanced apoptosis shown by elevated caspase‐3 levels in the desensitized group (Zhang et al., 2012). Earlier studies using systemic capsaicin pretreatment indicated that chemosensitive afferent nerves confer cardioprotective effects since prior to chemo‐denervation by capsaicin impaired post‐ischaemic recovery was shown in isolated rat hearts (Ustinova, Bergren, & Schultz, 1995) and an increase in the size of the myocardial infarction was found in an in vivo porcine model of acute myocardial infarction (AMI; Kallner, 1998). However, in the above‐mentioned studies, the presence of sensory neuropathy was not verified. The cardioprotective effect conferred by capsaicin‐sensitive afferents appeared to be mediated through activation of the TRPV1 receptor which in turn induces release of substance P and CGRP from sensory nerves. The role of the TRPV1 receptor in cardioprotection was supported by the finding that post‐ischaemic recovery was impaired in TRPV1 knockout mice (Wang & Wang, 2005). Furthermore, administration of an endogenous vanilloid, N‐oleoyl dopamine, protected the ex vivo isolated mouse heart from ischaemia–reperfusion injury by activating the TRPV1 receptor (Zhong & Wang, 2008).

In contrast, most recently, selective chemical ablation of TRPV1 positive neurons in the stellate ganglion by intra‐ganglionic injection of resiniferatoxin has been shown to decrease the incidence of severe ventricular arrhythmias in dogs subjected to 60‐min coronary ligation (Zhou et al., 2019). This study suggests a pro‐arrhythmic role of TRPV1 positive neurons in the sympathetic nervous system innervating the heart.

## 5 CARDIOPROTECTION IN THE PRESENCE OF SENSORY NEUROPATHY

Since neuropathy manifests in the clinical scenario in a complex manner, it is hard to separately characterize the sensory neuropathy components of a pathology. Human data are mostly related to different polyneuropathy conditions or cardiac autonomic neuropathy. However, the results of some animal models specifically mimicking sensory neuropathy revealed a role for sensory nerves in several mechanisms of cardioprotection. An excellent review was recently published about the function of intact cardiac innervation in cardioprotection by remote ischaemic conditioning (Hausenloy et al., 2019), focusing on diabetic neuropathy in remote conditioning. Therefore, in the present review, we have focused on myocardial ischaemia/reperfusion injury and cardioprotection in the presence of a wide range of co‐morbidities, where the deterioration of sensory neural system has been shown to be involved in these pathologies. Cardioprotection (local and remote) can be classified at three different levels which are stimulus, transfer and target levels (Kleinbongard, Skyschally, & Heusch, 2017). Both sensory and autonomic nerves might be involved in all of these three levels. The complex actions of sensory and autonomic nerves involving higher CNS regions, such as the spinal cord or brainstem, have been reviewed in detail (Kleinbongard et al., 2017). However, the current knowledge on the specific contribution of sensory nerves to cardioprotection is not sufficient precise to classify their diverse actions at these three levels of cardioprotection.

More than two decades ago, we showed that systemic desensitization of the capsaicin‐sensitive afferents by high‐dose capsaicin pretreatment in rats abolished the protective effect of pre‐conditioning evoked by rapid ventricular pacing in isolated hearts with coronary occlusion and reperfusion (Ferdinandy et al., 1997). Several publications reported that ischaemic pre‐conditioning‐induced cardioprotection is attenuated in animal models of Type 2 diabetes, when diabetic neuropathy is present (extensively reviewed elsewhere, Whittington, Babu, Mocanu, Yellon, & Hausenloy, 2012). Furthermore, the cardioprotective effect of patients' serum taken after cycles of BP cuff inflation/deflation of the arm was only detected in cases of healthy subjects and diabetics without neuropathic complications, when these serum samples were applied to the isolated rabbit hearts with ischaemia–reperfusion injury (Jensen, Stottrup, Kristiansen, & Botker, 2012). The critical role of TRPV1 receptors in ischaemic pre‐conditioning was also demonstrated in experiments showing that deletion of the TRPV1 gene impaired pre‐conditioning‐induced cardioprotection, the protection being mediated by the release of substance P and CGRP from sensory nerves (Zhong & Wang, 2008). The pivotal role of TRPV1 receptor activation is strongly supported by the results that TRPV1 knockout animals show impaired post‐ischaemic recovery (Wang & Wang, 2005). This combined experimental data unequivocally indicates that activation of cardiac capsaicin‐sensitive afferents confer potent cardioprotective effect on the myocardium, through the release of the above neuropeptides. Furthermore, nerve growth factor has been shown to be critical for up‐regulation of TRPV1 receptors in primary sensory neurons from adult rats (Winter, Forbes, Sternberg, & Lindsay, 1988), in particular those containing CGRP. The role of TRPV1 receptor in cardioprotection was supported by the finding that reperfusion injury of diabetic mice hearts was attenuated after nerve growth factor administration up‐regulated TRPV1 receptor expression (Zheng et al., 2015). Although these results clearly demonstrate the critical role of capsaicin‐sensitive afferent nerves and their TRPV1 receptors in cardioprotection against ischaemic injury, a potential role of TRPV1 receptors expressed by rat cardiomyocytes (Qi et al., 2015; Zvara et al., 2006) has not been considered. Data about the involvement of TRPA1 channels in cardioprotection are controversial. Activation of TRPA1 by optovin led to enhanced survival rate of cardiac myocytes subjected to simulated ischaemia and reperfusion (Lu, Piplani, McAllister, Hurt, & Gross, 2016). However, in vivo coronary occlusion/reperfusion in TRPA1 knockout mice resulted in a reduction of myocardial infarct size (Conklin et al., 2019).

Both substance P and CGRP have been shown to participate in sensory nerve‐mediated cardioprotection. Myocardial ischaemia is associated with a decrease in the cardiac level of substance P and in turn, administration of substance P reduced ischaemic myocardial damage in Langendorff‐perfused hearts of rats subjected to systemic capsaicin pretreatment (Ustinova et al., 1995). Experimental evidence indicates that CGRP released from capsaicin‐sensitive cardiac afferents also induces cardioprotection against ischaemic myocardial damage in pig and rat acute myocardial injury models (Kallner, 1998; Li, Xiao, Peng, & Deng, 1996). The role of CGRP and NO in the pacing‐induced and capsaicin‐sensitive nerve‐mediated cardioprotection has been previously demonstrated (Ferdinandy, 2009; Ferdinandy et al., 1997). Cardioprotective effect of remote pre‐conditioning induced by direct stimulation of sensory nerves is mediated by the release of cardioprotective humoral factors, including sensory neuropeptides and NO. This humoral response was abolished by topical DMSO treatment damaging the sensory nerves and also by intra‐arterial NO‐donor treatment applied prior to stimulating the sensory nerves with topical capsaicin treatment in an in vivo remote ischaemia/reperfusion rabbit model (Redington et al., 2012). Further evidence for the neural component of remote ischaemic pre‐conditioning is that hexamethonium, a selective ganglionic blocker, can abolish the cardioprotective protective effects of short mesenteric artery occlusion‐triggered adenosine release in an in vivo rat model of acute myocardial infarction (Liem, Verdouw, Ploeg, Kazim, & Duncker, 2002). However, the latter study has not investigated the role of sensory nerves in its remote ischaemic pre‐conditioning model. Recently, it has been reported that somas of sensory neurones may release exosomes (a type of extracellular vesicles) containing microRNAs and other factors by which they can regulate cell–cell communication (Simeoli et al., 2017).

## 6 CO‐MORBIDITIES SIMULTANEOUSLY AFFECTING THE SENSORY NERVES AND THE HEART

In this section, we describe several disease states leading to sensory neuropathy and ischaemic or other cardiac pathologies simultaneously. We summarize the main features of each disease, provide human data in relation to sensory neuropathy and cardiac symptoms and finally, show preclinical research models for sensory neuropathy of different aetiologies (see also Table 1). The available animal models are mainly rodent models with several limitations, but they can be suitable to investigate how sensory nerves influence the ischaemic myocardium. Large animal models for neuropathies are scarce in the literature, but some canine models for primary sensory neuropathies have recently been developed (Correard et al., 2019).

**TABLE 1:** Primary and secondary sensory neuropathies and their associated effects on cardiovascular function

Pathologies affecting cardiac sensory neurons		Disease	Clinical cardiac symptoms	References	Available animal models of the disease	References
Primary neuropathies	Inherited	Charcot–Marie–Tooth disease	Noncompaction cardiomyopathy, increased incidence of arrhythmias, and embolic complications	Eltawansy et al., 2015	CMT1: PMP22 overexpressed transgenic rodents	Huxley et al. 1998 Sereda et al. 1996
		Familial dysautonomy (HSAN Type III)	Cardiovascular instability, postural hypotension, episodic hypertension, QT variability, and ECG abnormalities	Goldstein et al., 2008 Solaimanzadeh et al. 2008	*Ikbkap* conditional‐knockout mouse lines, *Ikbkap* ^*flox/flox*^ and *ikbkap* ^*δ20/flox*^	Dietrich et al., 2012 Ohlen et al., 2017
		Hereditary transthyretin‐mediated amyloidosis	Refractory cardiomyopathy, orthostatic hypotension, and arrhythmias	Adams et al., 2019 Lai et al., 2019	Mouse expressing human transthyretin V30M in a heat shock transcription factor 1 null background	Santos et al., 2010
		Friedreich's ataxia	Hypertrophic cardiomyopathy, atrial fibrillation, and ventricular tachycardia	Peverill et al., 2018	Conditional frataxin knockout mouse	Puccio et al., 2001
Secondary neuropathies	Metabolic	Diabetes mellitus (Type 1)	Diastolic cardiomyopathy and heart failure with reduced ejection fraction	Voulgari et al., 2010	Single high dose of STZ or multiple moderate dose of STZ	Bakovic et al., 2018 Kellogg, Converso, Wiggin, Stevens, and Pop‐Busui, 2009 Grise et al. 2016 Xuan et al., 2015
		Diabetes mellitus (Type 2)	Increased heart rate	Aune et al., 2017	High‐fat diet for 14 weeks and single dose of STZ (30 mg·kg^−1^)	Li et al., 2017
		Pre‐diabetes	N/A	N/A	Single low‐dose of STZ (20 mg·kg^−1^) and high fat diet	Koncsos et al., 2016
		Alcoholism	No characteristic cardiovascular involvement	Koike et al., 2003	Mouse model of chronic plus binge alcohol feeding‐induced ethanol intoxication	Matyas et al., 2016
	Autoimmune	Systemic lupus erythematosus	Left ventricular systolic and diastolic dysfunction	Chen et al., 2016	N/A	N/A
		Rheumatoid arthritis	Conduction disturbances and arrhythmias	Buleu et al., 2019 Seferovic et al., 2006	TNF‐α‐transgenic mouse, K/bxn mouse, Collagen‐induced arthritis/Collagen‐antibody‐induced arthritis in rats and mice, Zymosan‐induced arthritis in rat, and methylated BSA mouse model	Asquith, Miller, McInnes, and Liew, 2009 Choudhary et al., 2018
		Sjögren's syndrome	Lower heart rate and BP variability	Kovacs et al., 2004	Autoimmune regulator gene deficient (Aire^−/−^) mice	Chen et al., 2017
	Toxic	Paclitaxel, Vincristine, and Adriamycin	Degenerative morphological alterations in cutaneous C‐fibre	Boyette‐Davis, Xin, Zhang, and Dougherty, 2011; Dux et al., 1999; Siau et al., 2006	N/A	N/A
		Statins	N/A	N/A	N/A	N/A
		Antibiotics/antiviral agents	N/A	N/A	N/A	N/A
		Industrial/agricultural toxins	N/A	N/A	N/A	N/A
	Other	Vitamin deficiency	Atrial fibrillation and sinus tachycardia	Puntambekar et al., 2009	N/A	N/A
		Hypothyroidism	N/A	N/A	N/A	N/A
		Viral infections (HIV, Zika, and hepatitis C)	N/A	N/A	N/A	N/A
		Guillain–Barré sy.	Arrhythmias	Vucic, Kiernan, and Cornblath, 2009	N/A	N/A

*Note.* N/A indicates no available data in the literature.

### 6.1 Primary neuropathies: Hereditary disorders

There are several types of genetic disorders, which lead to sensory neuropathy and may be related to cardiovascular symptoms.

Charcot–Marie–Tooth (CMT) disease is the most frequently occurring hereditary sensory peripheral polyneuropathy caused by mutations affecting mainly myelinated Schwann cells (CMT1) or it is characterized by axonal degeneration (CMT2; Timmerman, Strickland, & Zuchner, 2014). In the extensive literature on this disease only a few case reports refer cardiac effects. In a recent paper, regarding CMT disease, a novel cardiomyopathy condition, non‐compaction cardiomyopathy (or ventricular hypertrabeculation), was described (Eltawansy, Bakos, & Checton, 2015), which is accompanied with high incidence of arrhythmias and embolic complications. Given the numerous mutations and genes associated with CMT, there are several animal models under development. One of them the CMT1A, which represents 70–80% of all CMT1 cases, is caused by a duplication in peripheral myelin protein 22 (*PMP22*) gene (Inoue et al., 2001). However, cardiac involvements have not been investigated in these models yet.

Other, less common hereditary sensory and autonomic neuropathies (HSANs) are classified into six major types (I–VI) and several further subtypes (Lynch‐Godrei, De Repentigny, Gagnon, Trung, & Kothary, 2018). Solid evidence of cardiac involvement in these diseases is lacking with one exception, familial dysautonomia (FD, HSAN III). This is a rare neurological disorder caused by a splice mutation in the inhibitor of κ light polypeptide gene enhancer in B‐cells kinase complex‐associated protein (IKBKAP) gene. Cardiovascular instability is the most striking feature of FD, which is characterized by postural hypotension and episodic hypertension, as well as tachycardia and decreased cardiac sympathetic innervation (Goldstein, Eldadah, Sharabi, & Axelrod, 2008). FD patients have an increased index of QT variability and decreased heart rate variability which is predictive of mortality. Further, multiple conventional and advanced electrocardiographic abnormalities suggest structural heart disease. Modelling of FD disease (HSAN III) in mouse has been carried out by several research groups. The development of a suitable mouse model was challenging due to the differences in man and mouse gene splicing and the impact of the disease‐causing T to C transition (Rubin & Anderson, 2017). However, during the development of these models, many promising therapeutic strategies and drug candidates have been identified (Yoshida et al., 2015). First attempts to generate a humanized FD mouse using bacterial artificial chromosome to deliver the full human *IKBKAP* gene containing the mutation (IVS20 + T → C) into the mouse genome were unsuccessful, because the created mouse model did not manifest any FD symptoms and was phenotypically normal (Hims et al., 2007). Using knockout approach was also ineffective, because the complete deletion of mouse *Ikbkap* results in early embryonic lethality (Chen et al., 2009). Dietrich et al. created two lines of mice carrying a conditional inactivation Ikbkap allele, *Ikbkap* ^*flox/flox*^ and *Ikbkap* ^*Δ20/flox*^. Both lines of mice manifested features of FD, including severe reduction in nociceptive (trkA/CGRP positive) neurons at late gestation, small stature, ataxic gait, loss of fungiform papillae, kyphosis or kyphoscoliosis (Dietrich, Alli, Shanmugasundaram, & Dragatsis, 2012). In the FD mouse models, in dorsal root ganglia mitochondria were depolarized and fragmented and ROS levels were significantly increased, which could validate the presence of neuropathy. Mitochondrial dysfunction is a major factor mediating the death of *Ikbkap* ^*−/−*^ neurons (Ohlen, Russell, Brownstein, & Lefcort, 2017). In the absence of *Ikbkap*, cardiac innervation is reduced, but other characteristic cardiac symptoms, which were observed in humans have not been investigated in these animal models.

Hereditary transthyretin amyloidosis (hATTR) due to the deposition of insoluble amyloid fibrils leads to sensory and autonomic neuropathies with simultaneous cardiac complications characterized by refractory cardiomyopathy, which develops due to left ventricular amyloid deposits (Adams, Koike, Slama, & Coelho, 2019). Over 130 mutations may cause hATTR that manifest as combined cardiac and neurologic symptoms. In a most recent retrospective clinical study (Lai et al., 2019), heart failure was found to be the major cause of death in hATTR patients. The authors revealed that the most sensitive prognostic marker for cardiac involvement were global longitudinal strain and mitral valve E/A ratio during echocardiographic analysis. Similarly, to hATTR, there are other types of amyloid deposits, for example gelsolin amyloidosis (also known as familial amyloidosis Finnish type), which may simultaneously affect the heart and peripheral nervous system, thereby leading to ventricular arrhythmias or atrioventricular blocks. However, these consequences derive from multi‐organ amyloid accumulation rather than directly from amyloid‐induced damage of the autonomic or sensory nerves (Lehmonen, Kaasalainen, Atula, Mustonen, & Holmstrom, 2019). The attempt to create a successful animal model for hATTR started around 30 years ago (Shimada et al., 1989) and even the ideal model that replicates all human characteristics of the disease is still has not been obtained. In 2010, Santos Fernandes, and Saraiva created a mouse model expressing human transthyretin V30M mutation in a heat shock transcription factor 1 null background, which has been used several times. Santos et al. validated the chronic neurodegeneration in dorsal root ganglia and sciatic nerves, but not early pain symptoms of hATTR, and there was no data on cardiac involvement.

Friedreich's ataxia (FRDA) is a progressive neurodegenerative disease affecting mainly sensory neurons and nerves of the spinal cord and thereby leads to severe ataxia, nystagmus and other cerebellar symptoms. FRDA caused by an autosomal recessive mutation, which leads to a GAA repeat expansion in intron 1 in the gene of the mitochondrial protein frataxin (Durr et al., 1996). FRDA also involves cardiac disturbances such as increased LV wall thickness, reduced chamber size and finally hypertrophic cardiomyopathy leading to heart failure with preserved ejection fraction (HFpEF; Peverill, Donelan, Corben, & Delatycki, 2018). Impaired myocardial perfusion in an FRDA patient was described to be the result of microvascular abnormality without epicardial coronary artery disease (Raman, Dickerson, & Al‐Dahhak, 2008). The occurrence of atrial fibrillation and ventricular tachyarrhythmias is also common, which eventually increases cardiac mortality (Zipse & Aleong, 2016). Association between acute myocardial infarction and FRDA disease was found only in two case reports (Sharma, Kiyokawa, Kim, Lee, & Kasuya, 2002). In 2001, Puccio et al. were the first to create mutant mice through conditional gene targeting (frataxin‐deficient line) to evaluate treatment strategies for FRDA. When frataxin gene is ablated in brain (neural tissues) and heart, the mice demonstrate a severe disorganization of cardiomyocytes, dilated cardiomyopathy, very short life span, ataxia and loss of proprioception in behavioural studies, such as the rotarod.

Fabry or Anderson–Fabry disease is an X‐chromosome‐linked hereditary lipid storage disease caused by various mutations of the human GLA gene resulting in a decreased expression and activity of the lysosomal α‐galactosidase A (AGAL). Reduced enzymatic activity affects the degradation of the complex glycosphingolipid/globotriaosylceramide (also known as Gb3 or CD77). Prevailing effect of the impaired GSL catabolism is peripheral neuropathy affecting neurons of autonomic ganglia and Aδ‐ and C‐fibre sensory ganglion cells (see for review Politei et al., 2016). Small fibre neuropathy is characterized by pathological pain, impaired thermosensation and acroparaesthesia, as well as autonomic symptoms of reduced sweating (hypohydrosis), reduced heart rate variability and orthostatic hypotension (Politei et al., 2016). Cardiac manifestations of the disease include concentric left ventricular hypertrophy leading ultimately to cardiac failure, conduction block and ventricular arrhythmias or tachycardia (Baig et al., 2019), which are mainly accounted for by direct impairments of cardiomyocytes and coronary blood vessels. Recently, an early impairment of cardiac innervation has been demonstrated by SPECT studies showing gradual elimination of the sympathetic nerves innervating the left ventricle in patients affected by Fabry disease (Imbriaco et al., 2017). Similar abnormalities in sensory and autonomic functions have been observed in an animal model of the disease (Hofmann et al., 2018).

### 6.2 Secondary neuropathies: Diabetes

Diabetes mellitus is likely the most frequent metabolic disease that affects peripheral nervous system, especially sensory nerves. Diabetic patients with sensorimotor neuropathy develop clear signs of diffuse autonomic impairment affecting both the visceral sensory system and cardiac autonomic nervous systems (Softeland et al., 2014). Diabetes‐associated damage of sensory nerves occurs simultaneously with damage of autonomic nerves. Therefore, in this chapter, we discuss the presentation of cardiovascular autonomic neuropathy (CAN) instead of sensory neuropathy in diabetes mellitus. Cardiovascular autonomic neuropathy damages autonomic nerve fibres causing abnormalities mainly in heart rate and vascular dynamics (Agashe & Petak, 2018).

Cardiovascular autonomic neuropathy is a common complication of diabetes resulting in reduced ejection fraction and diastolic cardiomyopathy (Voulgari, Papadogiannis, & Tentolouris, 2010). It has been shown (Chessa et al., 2002) that a decrease in heart rate variability (HRV), which is an indicator of the reduced influence of parasympathetic efferent activity on the heart, is one of the earliest signs of cardiovascular autonomic neuropathy and is correlated with the duration of hyperglycaemia and the degree of glycaemic control in children with insulin dependent diabetes. Type 1 diabetes can cause left ventricular sympathetic dysinnervation with proximal hyperinnervation resulting in severe myocardial perfusion retention and left ventricular dysfunction (Stevens et al., 1998). Hypoglycaemia itself can induce QT interval prolongation, which is one of the major predictor of arrhythmias originated from both sympatho‐adrenal activation and a lowered serum potassium level (Lee et al., 2004). Resting tachycardia is not a specific sign of CAN, however increased heart rate enhanced all‐causes of mortality in Type 2 diabetic patients, as shown by a recent meta‐analysis (Aune et al., 2017). Diagnosis of cardiovascular autonomic neuropathy is still not conclusive, as some diagnostic symptoms are not highly specific for cardiovascular autonomic neuropathy (Spallone, 2019). Cardiovascular signs like tachycardia, QT prolongation and orthostatic hypotension, frequently occur in association with cardiovascular autonomic neuropathy and therefore suggest cardiovascular monitoring in diabetic patents (Spallone et al., 2011). Toronto consensus recommended the use of cardiovascular autonomic reflex tests (Valsalva manoeuvre, heart rate response to deep breathing and standing, and orthostatic hypotension tests) for diagnosis of cardiovascular autonomic neuropathy (Spallone et al., 2011).

Several mechanisms have been revealed from the abundant number of animal models which could be link cardiovascular autonomic neuropathy and worsened cardiac function. However, here we present only some of these mechanisms from the extensive literature. In streptozotocin (STZ)‐induced diabetic rats, the left ventricle has a biphasic pattern of sensory innervation in different stages of diabetes (Bakovic et al., 2018). In early diabetes mellitus (between 2 weeks and 2 months after induction) an increase of neurofilament 200 kDa (NF200, a marker for sensory myelinated neurons) and tyrosine‐hydroxylase (TH, marker of the sympathetic fibres) protein content could be found in the left ventricular wall and in the interventricular septum compared to control animals. While in the later stages (between 6 and 12 months post‐diabetes mellitus induction), the above increases in the density of NF200 and TH immunoreactive fibres have disappeared (Bakovic et al., 2018). In experimental diet and STZ‐induced diabetic model of rats, the sensory nerve impairment (validated by tail flick latency test) significantly exaggerated myocardial damages and increased the infarct size and myocyte apoptosis (Li et al., 2017). Using STZ‐induced diabetes in Male Sprague–Dawley rats (Xuan et al., 2015), analysis of heart rate variability revealed a relative sympathetic hyperinnervation, which was accompínied with increased ventricular arrhythmogenesis in the diabetic group as compared to the control. However, looking at the immunohistochemistry of choline O‐acetyltransferase (ChAT) and L‐tyrosine hydroxylase (TH) positive nerve fibres as marker of parasympathetic and sympathetic nerve respectively, short‐term diabetes (3 months) resulted in myocardial parasympathetic denervation without significant sympathetic neural damage. By 6 months, sympatho‐parasympathetic imbalance had further developed. This time‐dependent neural denervation and heterogeneous innervation might cause relatively sympathetic hyperinnervation, which increases ventricular arrhythmogenesis. Interestingly in the prediabetic state of a diabetic rat model induced by the combination of low‐dose STZ and high‐fat diet, it was shown that the deterioration of diastolic function and sensory neuropathy occurs earlier than the manifestation of diabetes (Koncsos et al., 2016).

### 6.3 Secondary neuropathies: Autoimmune disorders

Most peripheral neuropathies accompanying autoimmune disorders affect the limbs, however cardiac involvement can also be present (Burakgazi & AlMahameed, 2016).

Systemic lupus erythematosus (SLE) is a complex heterogeneous autoimmune disease characterized by autoantibody production and immune complex deposition followed by damage to target tissues. Chen, Tang, Zhu, and Xu (2016) found in their meta‐analysis of 22 studies that patients with SLE develop left ventricular systolic and diastolic dysfunction. Cardiac MR imaging mainly shows vasculitis, myocarditis, and myocardial infarction in patients with SLE (Mavrogeni et al., 2018). Accordingly, many studies reported that SLE patients have much higher cardiovascular risk and higher in‐hospital mortality rate after acute myocardial infarction (Ke et al., 2019). SLE may lead to sensory neuropathy, which is limited to the CNS or the optic nerve (Dammacco, 2018), but according to our knowledge there are no available data on sensory neuropathy affecting cardiac nerves in SLE patients. Although there are several animal models, none of them are relevant to the association between sensory neuropathy and the heart.

Rheumatoid arthritis (RA) is a chronic autoimmune disease leading to synovial inflammation and resulting in swelling and pain in and around the joints. Rheumatoid arthritis also affects the cardiovascular system including myocardium. Conduction disturbances and arrhythmias could be signs of cardiac involvement of sensory nerve impairment due to rheumatic diseases (Seferovic et al., 2006). Buleu, Sirbu, Caraba and Dragan, (2019) found that cardiac manifestations of systemic inflammation can occur frequently with different prevalence in rheumatic diseases and it can affect the myocardium, cardiac valves, pericardium, conduction system and arterial vasculature. However, the presence of sensory neuropathy was not tested in any of the above‐mentioned studies with rheumatoid arthritis patients. Nevertheless, a recent case report showed a silent myocardial infarction in a rheumatoid arthritis patient with diagnosed cardiac autonomic neuropathy (Unnikrishnan, Jacob, Anthony Diaz, & Lederman, 2016). Animal models for rheumatoid arthritis include induced arthritis models, such as genetically manipulated, the spontaneous arthritis models, such as the TNF‐α‐transgenic mouse, K/BxN mouse and the collagen‐induced arthritis, such as collagen‐antibody‐induced arthritis, zymosan‐induced arthritis and the methylated BSA model (Choudhary, Bhatt, & Prabhavalkar, 2018). In the animal models of rheumatoid arthritis, sensory neuropathy in the heart has not been investigated. The effect of long‐term inflammation in the collagen antibody‐induced arthritis mouse model, Pironti et al. showed that there was long‐term consequences of molecular remodelling on the contractile function of the heart. They found that rheumatoid arthritis contributes to the development of heart failure, cardiomyopathy and contractile dysfunction, fibrosis and reduced left ventricular fractional shortening compared to control (Pironti et al., 2018).

Sjögren's syndrome is a chronic autoimmune disorder that affects mainly the lacrimal and salivary glands and could cause peripheral neuropathy and dry eye. Signs of cardiovascular autonomic nervous system dysfunction was detected in majority of patients affecting heart rate, BP variability, spontaneous baroreflex sensitivity and cardiovascular reflexes (Kovacs et al., 2004). Primary Sjögren syndrome may cause severe arteriosclerosis, development of ischaemic heart disease and occurrence of sudden cardiac death (Inoue et al., 2008). Like any other complex human disease, Sjögren's syndrome has no single animal model which could replicate all aspects of the disease. Therefore, animal models of Sjögren's syndrome have a huge variability as there are spontaneous, genetically engineered and experimentally induced models. The autoimmune regulator (Aire) gene‐deficient mice develop spontaneous, CD4^+^ T cell‐mediated exocrinopathy, which leads to dry eye that is associated with loss of nerves innervating the cornea and lacrimal gland. Peripheral neuropathies characteristic for Sjögren's syndrome is tightly linked with the underlying immunopathological mechanism, and Aire^−/−^ mice provide an excellent tool to explore the interplay between Sjögren's syndrome associated immunopathology and peripheral neuropathy (Chen et al., 2017). However, cardiac effects in the above mice model have not been investigated yet.

### 6.4 Secondary neuropathies: Drug‐induced and toxic neuropathies

There are several xenobiotics including drugs, toxins and chemicals, which may cause sensory neuropathy. A detailed list of these compounds is available in a recent review by Karam and Dyck, who distinguished different appearance and manifestation of neuropathies caused by the such compounds (Karam & Dyck, 2015). Since treatment with the specific compound leads to the same deleterious effect on sensory nerves as in humans, detailed descriptions of these models would exceed the scope of the present review.

Anticancer drugs including taxanes, platinum derivatives and vinca alkaloids possess the most abundant literature related to neuropathies, which are predominantly sensory in type. Indeed, morphological studies show degenerative alterations in cutaneous C‐fibre innervation following the administration of paclitaxel, vincristine (Siau, Xiao, & Bennett, 2006) and anthracyclines (adriamycin/doxorubicin; Boros et al., 2016) similar to that seen after capsaicin treatment (Dux, Sann, Schemann, & Jancso, 1999). Bortezomib, a proteasome inhibitor used for the treatment of multiple myeloma and thalidomide, an immunomodulatory drug against Mycobacterium subspecies, are also able to evoke severe sensory neuropathy characterized by painful paraesthesia due to the peripheral loss of large myelinated fibres (Karam & Dyck, 2015). In contrast to anthracyclins or taxanes, only the occasional but not severe or significant cardiotoxicity has been shown to be caused by either bortezomib or thalidomide (Reneau et al., 2017).

Among cardiovascular drugs, the cholesterol‐lowering statins (simvastatin, pravastatin and fluvastatin) seem to have significant risk factors for the development of primary sensory neuropathy (Jones et al., 2019). However, only a few clinical trials or meta‐analysis studies have identified statin‐related peripheral neuropathy, which was predominantly of the sensory type (summarized in the review of Jones et al., 2019), and the mechanism is also largely unknown. It is supposed to be associated with energy depletion of neurons due to decreased ubiquinone synthesis. However, their well‐known cardioprotective and anti‐atherosclerotic effects are far outweigh the sporadic appearance of statin‐derived sensory neuropathy. Also, the anti‐arrhythmic drug, amiodarone, in high dose and over a long duration has a risk of causing sensory and motor deficits in patients (Jones et al., 2019).

There are a couple of antibiotics (nitrofurantoin, linezolid, ethambutol and chloramphenicol) which develop neuropathy during chronic application. However, most of them induce only a slight sensory rather than other types of neuropathies (see for review Karam & Dyck, 2015). The one exception is the group of antiretroviral agents (nucleoside analogue reverse transcriptase inhibitors e.g. zalcitabine and stavudine), which may lead to severe distal sensory neuropathy accompanied by neuropathic pain.

Beyond medical treatments, it is worth mentioning that agriculturally or industrially used compounds, such as organophosphates and vacor (pyrinuron), as well as dimethylaminopropionitrile (Staff & Windebank, 2014), may lead to the development of severe sensory neuropathy (see for review Karam & Dyck, 2015). However, their cardiac effects due to sensory neuropathy are hard to distinguish from their severe systemic actions, for example increased vagal tone due to cholinesterase inhibition (organophosphates) or hyperglycaemic ketoacidosis in case of vacor.

It is well known that chronic ethanol abuse leads to sensory and also motor neuropathy. Specifically, alcohol‐related neuropathy presents with a slowly progressive, sensory‐dominant symptoms, while thiamine deficiency causes an acutely progressive (<1 month in 56%), primarily motor‐dominant features with the loss of ambulation (Koike et al., 2003). Moreover, it has been shown that ethanol intoxication‐induced sensory neuropathy alone does not lead to heart failure, only when alcohol abuse was combined with vitamin B1 (thiamine) deficiency (Koike et al., 2003). However, chronic ethanol abuse leads to enhanced cardiac mitochondrial oxidative stress accompanied with myocardial dysfunction, as demonstrated recently by Matyas et al. (2016) using a mouse model of chronic plus binge alcohol feeding‐induced ethanol intoxication. Thus, direct cardiac effects of ethanol intoxication show a more pronounced cardiotoxicity than that of indirectly caused by sensory nerve injury.

There are several further toxins including inorganic compounds as thallium, arsenic or lead, as well as biotoxins such as tetrodotoxin, diphtheria or tick paralysis toxin, which may contribute to the development of sensory neuropathy. However, they cause development of complex multi‐organ injuries including the heart. Therefore, their discussion would exceed the scope of the present review and are discussed in detail elsewhere (Karam & Dyck, 2015).

It is not known how sensory neuropathy may contribute to the “hidden cardiotoxicity” (a novel concept, i.e. cardiotoxicity seen only in the diseased heart) of some drugs and toxins, including, for example, statins (Ferdinandy et al., 2019).

### 6.5 Secondary neuropathies: Vitamin and cofactor deficiencies

Beyond the most common metabolic disorders such as diabetes and the toxic effects of certain drugs, several other factors or diseases can lead to the development of sensory neuropathies. As we previously discussed in ethanol intoxication section, thiamine (vitamin B1) deficiency is also an aetiological factor for sensory (and motor) neuropathy accompanied with cardiovascular complications, based on the results of a study where over 2/3 of the enrolled patients with vitamin B1 deficiency developed signs of heart failure (Koike et al., 2003). The lack of cobalamin (vitamin B12) may cause primary sensory demyelination (Steiner, Kidron, Soffer, Wirguin, & Abramsky, 1988). Puntambekar, Basha, Zak, and Madhavan (2009) reported a case of a patient with vitamin B12 deficiency developing atrial fibrillation and sinus tachycardia due to sensory and autonomic neural dysfunction. However, other publications related to cobalamin deficiency do not refer to any cardiovascular symptoms. It has been shown that isolated vitamin E deficiency may lead to peripheral neuropathy, but it was not accompanied by ECG alterations (Puri, Chaudhry, Tatke, & Prakash, 2005). In contrary, excessive intake of pyridoxin (vitamin B6) also resulted in similar sensory neuropathy both in patients (Gdynia et al., 2008) and in a rat model (Windebank, Low, Blexrud, Schmelzer, & Schaumburg, 1985), but any signs of cardiac dysfunctions were not measured.

### 6.6 Secondary neuropathies: Others

Infections by human immunodeficiency virus (Karpul, McIntyre, van Schaik, Breen, & Heckmann, 2019), hepatitis C (Yoon et al., 2011) or Zika virus (Medina & Medina‐Montoya, 2017), as well as alteration of hormonal status such as hypothyroidism (Eslamian et al., 2011) have been shown to lead to sensory neuropathy. Guillain–Barré syndrome is a rare disease when the immune system starts to attack the nervous system primarily causing acute inflammatory demyelinating polyradiculoneuropathy (Burakgazi & AlMahameed, 2016). The autonomic and cardiovascular involvement in Guillain–Barré syndrome involve mainly the autonomic fibres (Burakgazi & AlMahameed, 2016). However, in all the above‐mentioned conditions, the aetiological role of sensory dysfunction in the cardiovascular complications is not obvious; cardiac pathology develops directly in response to the primary disease (i.e. infections or hormonal disorders).

## 7 SENSORY NEUROPATHY AND ITS PRECLINICAL MODELS

Sensory neuropathy denotes diseases of the sensory nerves in which either the axon or the myelin‐forming cells are dysfunctional due to metabolic, toxic, infectious or genetic causes, which results in several clinical symptoms like hyperalgesia, allodynia or the opposite, reduced ability to sense pain or extreme temperatures and paraesthesias (Ochoa, 1995). There are plenty of pathologies leading to sensory neuropathies (see more details on animal models above), although isolated sensory neuropathy can be achieved experimentally by the systemic application of capsaicin or its ultrapotent analogue, resiniferatoxin.

Neurochemical and histochemical studies have demonstrated a remarkable reductions in substance P and CGRP levels in sensory nerves (Gamse et al., 1981), including cardiac nerves (Ferdinandy et al., 1997), after systemic capsaicin treatment, also referred to as capsaicin desensitization or selective sensory chemodenervation (Dux et al., 1999). This is a unique and selective experimental tool that is used to mimic sensory neuropathies induced by metabolic disorders or drug treatments allows the investigation of the role of peptidergic capsaicin‐sensitive sensory nerves in a variety of pathophysiological mechanisms.

Resiniferatoxin is an ultrapotent analogue of capsaicin, which also selectively acts on primary sensory neurons resulting in ultrastructural alterations and CGRP depletion. Resiniferatoxin does not change heart rate or BP nor does it provoke the pulmonary chemoreflex in the rat, the latter is the main limiting factor in capsaicin treatment (Szolcsanyi, Szallasi, Szallasi, Joo, & Blumberg, 1990). This suggests a further advantage in using resiniferatoxin to investigate capsaicin‐sensitive neural pathways (Szolcsanyi et al., 1990). In isolated rat cardiac mitochondria, resiniferatoxin caused concentration‐dependent decrease in oxygen consumption and lowered mitochondrial membrane potential (Athanasiou et al., 2007), since TRPV1 channels are located in intracellular and mitochondrial membranes as well.

Resiniferatoxin application has beneficial effects on cardiac and autonomic dysfunction in rats with myocardial infarction‐induced chronic heart failure. Epicardial application of resiniferatoxin in rats 9–11 weeks after the myocardial ischaemia interrupts already activated cardiac sympathetic afferents, thereby reducing a major source of sympatho‐excitation in chronic heart failure. This experimental result shows the importance and therapeutic potential of targeted desensitization of cardiac sympathetic afferent reflex in ventricular remodelling after myocardial infarction (Wang, Wang, Cornish, Rozanski, & Zucker, 2014). Furthermore, epicardial resiniferatoxin application lowered diastolic BP both at daytime and night‐time with less effect on systolic BP in chronic heart failure rats compared to the sham group. Cardiac sympathetic afferent reflex activation physiologically causes significant increase in cardiac contractility and output (Wang, Rozanski, & Zucker, 2017). In the rat model of transverse aortic constriction‐induced cardiac remodelling resiniferatoxin pretreatment improved haemodynamic data (reduced left ventricular end‐diastolic pressure) and also prevented cardiac hypertrophy, fibrosis and apoptosis. Focal chemo‐ablation of TRPV1^+^ neurons in the spinal cord protects the heart from pressure overload‐induced cardiac remodelling and cardiac dysfunction, which could be a promising novel therapeutic treatment against cardiac hypertrophy and diastolic dysfunction (Wang, Wu, et al., 2019).

## 8 SIMULTANEOUS THERAPEUTIC OPTIONS FOR THE ISCHAEMIC HEART IN SENSORY NEUROPATHY

There is no available drug treatment for indications of sensory neuropathy approved by the Federal Drug Agency or the European Medicines Agency. In most cases, the treatment of sensory neuropathy consists of the symptomatic pharmacological treatment of neuropathic pain related to diabetes‐ or chemotherapy‐induced neuropathy. Gwathmey and Pearson (2019) summarized their findings on the above therapies and classified them in three levels (A to C, where A is the most preferable) according to the strength of evidence for efficacy. According to the above‐mentioned review, at Level A, the treatment of neuropathic pain can be treated with the following drugs, gabapentin, pregabalin, venlafaxine, duloxetine, tricyclic antidepressants (e.g. amitriptyline), tramadol and oxycodone, at Level B valproate, dextromethorphan, capsaicin and botulinum toxin should be used, while at Level C carbamazepine. Since there is no causal treatment for sensory neuropathy, there is an utmost need for novel therapeutic targets focusing on preservation of physiological functions of nerves/neurons and cardiac myocytes simultaneously. In Clinicaltrials.gov database, 56 studies can be found by the term “sensory neuropathy.” In 20 of these studies, the study protocol includes pharmacological approaches to improve the primary disease or to alleviate pain symptoms, however none of them relates to cardioprotection.

One of the most promising novel drug candidates in experimental phase is BGP‐15 that could improve hereditary sensory and autonomic neuropathy Type III (HSAN III, also called FD). FD is a genetic disorder caused by a single base substitution in the IKBKAP gene resulting in disrupt development of the peripheral nervous system, which also affects sensory nerves. This condition can have fatal consequences resulting from functional and electrophysiological instability, respiratory dysfunction and/or sudden death during sleep (Norcliffe‐Kaufmann, Slaugenhaupt, & Kaufmann, 2017). Ohlen et al. (2017) demonstrated by using an *Ikbkap/Elp1* conditional‐knockout mouse model that daily injections of BGP‐15 (O‐(3‐piperidino‐2‐hydroxy‐1‐propyl)nicotinic amidoxime), a cardioprotective compound developed by a Hungarian biotech company in late 1990s, significantly improved cardiac innervation and prevented the death of *Ikbkap* ^*−/−*^ neurons (Table 2). BGP‐15 was basically developed against PARP as an insulin sensitizer drug, but it has been shown that it protects isolated rat hearts against ischaemia/reperfusion injury (Szabados, Literati‐Nagy, Farkas, & Sumegi, 2000). Recently, BGP‐15 has been shown to prevent the impairment of sensory nerves conduction velocity in cisplatin‐ or taxol‐induced peripheral neuropathy in rats (Bárdos et al., 2003). More recently, BGP‐15 was applied against diabetic cardiomyopathy in a spontaneous diabetic Goto‐Kakizaki rat model (Bombicz et al., 2019). The BGP‐15‐treatment considerably enhanced diastolic function and improved Tei‐index in Goto‐Kakizaki rats, by affecting the SERCA/phospholamban pathway (Bombicz et al., 2019). Beneficial effects of BGP‐15 have been shown in models of diabetes, muscular dystrophy, HSAN and also in heart failure, where it has affected chaperones, contractile and mitochondrial proteins, as well as SERCA (Gehrig et al., 2012; Ohlen et al., 2017; Sapra et al., 2014).

**TABLE 2:** Novel and promising therapeutic approaches to improve sensory neuropathy and to decrease infarct size/preserve cardiac function

Drug/agent	Aetiology	Therapeutic target	Achieved improvement	References
Therapies approved by FDA/EMA				
α‐Lipoic acid	Diabetes‐associated CAN	Antioxidant therapy to decrease diabetic oxidative stress	Improvement of diabetes‐induced CAN symptoms; reduction in myocardial infarct size	Deng et al., 2013; Ziegler et al., 1997
α‐Tocopherol	Diabetes‐associated CAN	Antioxidant therapy to decrease diabetic oxidative stress	Improvement of diabetes‐induced CAN symptoms; decreased infarct size and improved left ventricular function	Wallert et al., 2019; Ziegler et al., 1997
Therapies under preclinical or clinical investigation				
BGP‐15	HSAN Type III (Familial dysautonomia)	IKBKAP gene	Protects isolated rat hearts against ischaemia/reperfusion injury; significantly improves cardiac innervation, prevents the death of Ikbkap^−/−^ neurons	Bombicz et al., 2019; Ohlen et al., 2017; Szabados et al., 2000
Aldose reductase inhibitors (ranirestat, zopolrestat, and epalrestat)	Diabetes‐associated CAN	Aldose reductase, blocks polyol pathway	Reduce myocardial infarct size in Type 1 diabetes; improvement of sensory nerve conduction velocity in both Type 1 or Type 2 diabetic patients	Annapurna et al., 2008; Sekiguchi et al., 2019
PDE5 inhibitors (sildenafil, vardenafil, and tadanafil)	Type 2 diabetes‐induced sensory neuropathy	PDE type 5	Reduction in infarct size in both normal and Type 2 diabetic rodents; improve diabetes‐induced HFpEF symptoms	Varma et al., 2012; Wang et al., 2016, Matyas et al., 2017
Nerve growth factor (NGF)	Type 1 or Type 2 diabetes	Facilitates TRPV1 development	Attenuated reperfusion injury of diabetic rat hearts; Adenovirus‐mediated NGF gene delivery prevented sensory neuropathy in bone marrow and restored blood flow in limb ischaemia	Dang et al., 2019; Zheng et al., 2015

CAN; cardiovascular autonomic neuropathy.

To find efficacious treatment for diabetes mellitus‐associated sensory neuropathy is critical since the incidence of diabetes and thereby the appearance of sensory neuropathy is rapidly increasing worldwide. As we discussed above, cardiovascular autonomic neuropathy is a frequent complication of diabetes. It was shown that there is a prognostic value of cardiovascular autonomic neuropathy for cardiovascular mortality and morbidity in both Type 1 and Type 2 diabetes (Spallone, 2019). Besides the association with both fatal‐ and non‐fatal cardiovascular events, cardiovascular autonomic neuropathy also showed strong correlation with glycaemic control (Pop‐Busui et al., 2017). Prevention and existing treatment of cardiovascular autonomic neuropathy include lifestyle modifications and strict control of blood glucose level (involving oral antidiabetic drugs or insulin therapy if needed). However, the pathogenesis‐based pharmacological treatment consists of antioxidant therapy such as α‐lipoic acid and vitamin E (Table 2; Ziegler et al., 1997). α‐Lipoic acid has been recently shown to reduce myocardial infarct size when administered 30 min before coronary occlusion in rats (Deng et al., 2013). Most recently, α‐tocopherol, the strongest antioxidant form of vitamin E, applied 2 hr prior to 60‐min coronary occlusion, immediately after reperfusion and twice per day for three consecutive days in mice, has been shown to decrease infarct size and improve left ventricular function 4 weeks after the development of acute myocardial infarction (Wallert et al., 2019). As an alternative option for improving sensory function in diabetic patients suffering from cardiovascular autonomic neuropathy is the inhibitors of aldose reductase (Table 2; e.g. ranirestat, zopolrestat and epalrestat). Ranirestat and zopolrestat are already tested in clinical phases and have been shown to improve sensory nerve conduction velocity in both Type 1 and Type 2 diabetic patients (Sekiguchi et al., 2019) and to stabilize or partially reverse left ventricular abnormalities in cardiovascular autonomic neuropathy patients (Fisher & Tahrani, 2017). Moreover, non‐selective inhibition of aldose reductase by suldinac has been shown to reduce myocardial infarct size in normal and also in STZ‐induced diabetic rats (Annapurna, Challa, Prakash, & Viswanath, 2008).

Recently, a novel cardiovascular and anti‐diabetic/anti‐neuropathic indication for phosphodiesterase type 5 (PDE5) inhibitors (e.g. sildenafil, tadalafil and vardenafil) have been intensively studied. PDE5 selectively hydrolyses the second messenger cGMP, thereby regulating its intracellular concentrations. Dysregulation of the cGMP‐dependent pathway plays a crucial role in various cardiovascular diseases such as erectile dysfunction and pulmonary hypertension (Tsai & Kass, 2009). Tadalafil has previously been shown to reduce infarct size in both normal and Type 2 diabetic rats and mice (Table 2; reviewed in Das, Durrant, Salloum, Xi, & Kukreja, 2015). Furthermore, recently, it has been shown that tadalafil improves sensory nerve conduction velocity in Type 2 diabetic db/db mice (Wang et al., 2016). Long‐term administration of vardenafil led to effective prevention of developing HFpEF characterized by increased myocardial stiffness and worsened diastolic function in Type 2 diabetic Zucker diabetic fatty rats. In the same study, vardenafil treatment also reduced the pathophysiological features of diabetes‐associated cardiomyopathy and restored the activity of cGMP–PKG axis by increasing myocardial and plasma cGMP levels (Matyas et al., 2017). Thus, PDE5 inhibition may simultaneously reduce myocardial infarct size, achieve improvement in Type 2 diabetes, one of the major causes of sensory neuropathy and recovers myocardium from HFpEF. However, the proportion of diabetes‐induced sensory neuropathy in the development of diabetic HFpEF still remains the subject of further investigations.

## 9 CONCLUSIONS AND FUTURE PERSPECTIVES

Abundant data from literature demonstrate that sensory nerves play pivotal roles in the physiological and pathological of the heart. Despite, in the last decades, research starting to focus on the role of cardiac innervation in cardiac pathologies and cardioprotection, little is still known on the mechanism and the effect of sensory neuropathy on cardiovascular morbidities, especially ischaemic heart disease and cardioprotection. Nevertheless, several promising therapeutic approaches have emerged recently which may have beneficial actions on myocardial ischaemic injury and simultaneously improve the symptoms of sensory neuropathy of different aetiologies.

### 9.1 Nomenclature of targets and ligands

Key protein targets and ligands in this article are hyperlinked in corresponding entries in http://www.guidetopharmacology.org, the common portal for data from the IUPHAR/BPS Guide to PHARMACOLOGY (Harding et al., 2018) and are permanently archived in the Concise Guide to PHARMACOLOGY 2019/20 (Alexander et al., 2019).

## CONFLICT OF INTEREST

P.F. is the founder and CEO of Pharmahungary, a group of R&D companies.
